# College Notes

**Published:** 1902-08

**Authors:** 


### COLLEGE NOTES.

The Denver College of Medicine and the Gross Medical College announce that they have united under the name of the Denver and Gross College of Medicine, medical department of the University of Denver.

The college will be located in the Haish building, corner Fourteenth and Arapahoe Streets, Denver, Col., heretofore the home of the Denver College of Medicine.

The teaching force will embrace the faculty and instructors of each school under the management of a board of trustees selected from each of the schools as follows: Dr. E. C. Rivers, president of the board; Drs. Thomas H. Hawkins, E. J. A. Rogers, S. G. Bonney, C. K. Fleming, W. A. Jayne, Robert Levy, Leonard Freeman and Henry Sewall.

The facilities and equipment of each school will be utilised in the new college and very material additions will be made thereto with the result that the college will offer a four-year graded course consisting of four sessions of eight months each. The laboratories in all departments are modern and will be greatly enlarged and thoroughly equipped.

The first two years of the course will be devoted to fundamental principles of the science of medicine and to laboratory work, and the last two years to the study of the practical branches of medicine and surgery, the application of laboratory methods in diagnosis and to clinical work by the students, which will form a prominent feature of the course.

The University of Buffalo, Medical Department, has issued its annual announcement while these pages are on the press. From it we learn that the 57th regular session will begin Monday, September 29, 1902.

